# Mobilome-driven antimicrobial resistance in a one health context: evidence and lessons from Africa

**DOI:** 10.3389/fvets.2026.1805112

**Published:** 2026-08-19

**Authors:** Zikora Kizito Glory Anyaegbunam, Yandev Doowuese, Chibuzor Kenneth Uwazie, Theophilus Ajanaobionye, Chinaka Chinwe Blessing, Emeter Nkoli Winifred, Onyekachi Philomena Okeke, Ifeanyi Elibe Mba

**Affiliations:** 1 Department of Microbiology, University of Nigeria, Nsukka, Nigeria; 2 Institute for Drug-Herbal Medicine-Excipient Research and Development, University of Nigeria, Nsukka, Nigeria; 3 Department of Microbiology, Federal University of Health Sciences, Otukpo, Nigeria; 4 Chemistry and Biochemistry Department, Western Kentucky University, Bowling Green, KY, United States; 5 Department of Adult Nursing, University of Salford, Salford, United Kingdom; 6 Department of Science Laboratory Technology, University of Nigeria, Nsukka, Nigeria; 7 Public Health Unit, Global Health Infectious Disease Institute, Nasarawa State University, Keffi, Nigeria; 8 Department of Biomedical Sciences, Augusta University, Augusta, GA, United States; 9 Department of Pharmaceutical Microbiology, Faculty of Pharmacy, University of Ibadan, Ibadan, Nigeria

**Keywords:** Africa, animal–environment interfaces, antimicrobial resistance, genomic surveillance, mobile genetic elements, mobilome, multidrug resistance, one health

## Abstract

Antimicrobial resistance (AMR) is one of the most urgent global health threats and is increasingly recognized as a One Health challenge driven by interactions among human, animal, and environmental reservoirs. Central to the emergence and dissemination of AMR across these interfaces are mobile genetic elements (MGEs), which form an interconnected mobilome capable of transferring resistance genes across bacterial taxa and ecological niches. These elements facilitate the accumulation and spread of multidrug resistance determinants and are shaped by co-selective pressures operating at the animal–environment–human interface. Despite their critical role, genomic surveillance of MGEs remains limited, particularly in high-burden regions such as Africa. This narrative review synthesizes evidence from published genomic surveillance studies, primarily whole-genome sequencing–based analyses, to examine the distribution and dynamics of AMR genes and MGEs across One Health interfaces. We highlight animal–environmental systems as major hotspots for mobilome-driven resistance dissemination and also evaluate key advances, methodological approaches, and persistent surveillance challenges and gaps specific across Africa. By integrating findings from diverse genomic studies, and highlighting key lessons and implementation gaps from One Health studies across Africa, this review underscores the need for coordinated One Health surveillance strategies to better capture mobilome dynamics and inform sustainable AMR control efforts.

## Introduction

AMR is a major global health threat that undermines the effectiveness of antibiotics and other antimicrobial agents. The emergence and spread of multidrug-resistant (MDR) and extensively drug-resistant (XDR) bacteria are driven largely by antimicrobial misuse and overuse in human medicine, animal production, and agriculture, particularly among Gram-negative pathogens associated with respiratory, bloodstream, and healthcare-associated infections (1–3). The burden of AMR is especially severe in low- and middle-income countries (LMICs), where weak healthcare systems, inadequate sanitation, limited laboratory capacity, and high infectious disease prevalence contribute to rapid dissemination of resistant organisms (4, 5). Despite advances in infection prevention, antimicrobial stewardship, and vaccination, fragmented surveillance systems and limited genomic monitoring continue to hinder effective AMR control in many high-burden settings (6, 7).

The complex ecology of AMR has reinforced the importance of the One Health framework, which recognizes the interconnectedness of human, animal, and environmental health systems. Resistant bacteria and AMR genes circulate across clinical, agricultural, food, and environmental interfaces through contaminated water systems, livestock production, wastewater discharge, and human-animal interactions. Consequently, AMR cannot be effectively addressed through isolated clinical or pharmaceutical interventions alone. Increasingly, integrated One Health surveillance programs are being adopted to improve cross-sectoral monitoring and response strategies, particularly in LMICs where environmental reservoirs of resistance remain under-characterized (8, 9). Initiatives such as the WHO Tricycle Project and regional surveillance networks in Africa have demonstrated the value of coordinated surveillance across humans, animals, and the environment, while also highlighting persistent challenges related to infrastructure, governance, and data harmonization (10, 11).

Central to AMR dissemination is the mobilome, the collection of MGEs that mediate horizontal gene transfer between bacteria. Plasmids, transposons, insertion sequences, integrative and conjugative elements (ICEs), integrons, and prophages facilitate the capture, assembly, and transfer of resistance determinants across bacterial populations and ecological niches. Conjugative plasmids are particularly important because they enable rapid transfer of AMR genes between diverse bacterial species and frequently interact with insertion sequences and transposons to form highly dynamic resistance platforms (12, 13). Similarly, prophages and phage-plasmids can disseminate clinically important resistance genes independently of direct cell-to-cell contact, expanding the range and persistence of AMR transmission networks (14). Increasing genomic evidence indicates that MGEs account for the majority of horizontally transferable resistance determinants and are key drivers of AMR evolution across One Health settings (15, 16).

Although AMR is widely recognized as a global crisis, the mechanisms underlying resistance exchange across human, animal, and environmental systems remain incompletely understood, particularly in Africa and other high-burden regions with limited genomic surveillance capacity. This narrative review addresses this gap by examining AMR through a mobilome-centered perspective, emphasizing MGEs as the primary mediators of resistance dissemination across One Health interfaces. Drawing on recent genomic and metagenomic studies from Africa, we evaluate the role of plasmids, integrons, transposons, insertion sequences, and related MGEs in shaping AMR transmission dynamics across clinical, agricultural, and environmental reservoirs. We further discuss the implications of these findings for integrated surveillance, policy development, and AMR mitigation strategies within resource-limited settings.

## Review methodology

This narrative review was developed through a targeted search of the scientific literature to identify studies examining the role of MGEs in the emergence and dissemination of AMR within a One Health framework, with particular emphasis on evidence from Africa. Literature searches were conducted in PubMed, Scopus, Web of Science, and Google Scholar. The searches covered papers published between 2000 to 2026. Search terms included combinations of keywords such as “mobilome,” “mobile genetic elements,” “antimicrobial resistance,” “resistance genes,” “horizontal gene transfer,” “whole-genome sequencing,” “genomic surveillance,” “One Health,” and “Africa.” Boolean operators (AND, OR) were applied where appropriate to refine search results and improve retrieval of relevant studies. Studies were considered eligible if they (i) investigated the role of MGEs (including plasmids, transposons, integrons, insertion sequences, prophages, or integrative and conjugative elements) in the emergence, maintenance, or transmission of AMR; (ii) reported evidence from animal. Humana, environmental, or integrated One Health settings; (iii) employed molecular or genomic approaches, including WGS, metagenomics, or other sequencing-based technologies, to characterize AMR determinants or MGEs; or (iv) described One Health surveillance strategies, governance models, or implementation experiences relevant to AMR control. Original research articles, systematic reviews, narrative reviews, and reports from recognized international organizations were considered where they contributed relevant evidence. Studies were excluded if they focused solely on antimicrobial resistance mechanisms unrelated to MGEs or horizontal gene transfer, lacked sufficient methodological detail, were duplicate publications, conference abstracts without full-text articles, editorials, commentaries, or publications not directly relevant to the objectives of this review. Only articles published in English were included. Additional relevant publications were identified through manual screening of reference lists from selected articles and pertinent review papers. Although this review synthesizes evidence from the global literature to provide a comprehensive overview of mobilome-mediated AMR dissemination, special emphasis was placed on studies conducted in Africa. This focus reflects the continent’s growing AMR burden, limited genomic surveillance capacity, and unique One Health challenges. In addition, the review includes a dedicated section, “Implementing One Health in Africa: Governance Models, Community Delivery, and Lessons Learned,” which examines practical experiences and implementation strategies from diverse African settings. This section was incorporated to properly highlight context-specific lessons, identify implementation challenges, and explore opportunities for strengthening One Health approaches to AMR surveillance and control across the continent.

## AMR through a one health lens

**Figure 1:**
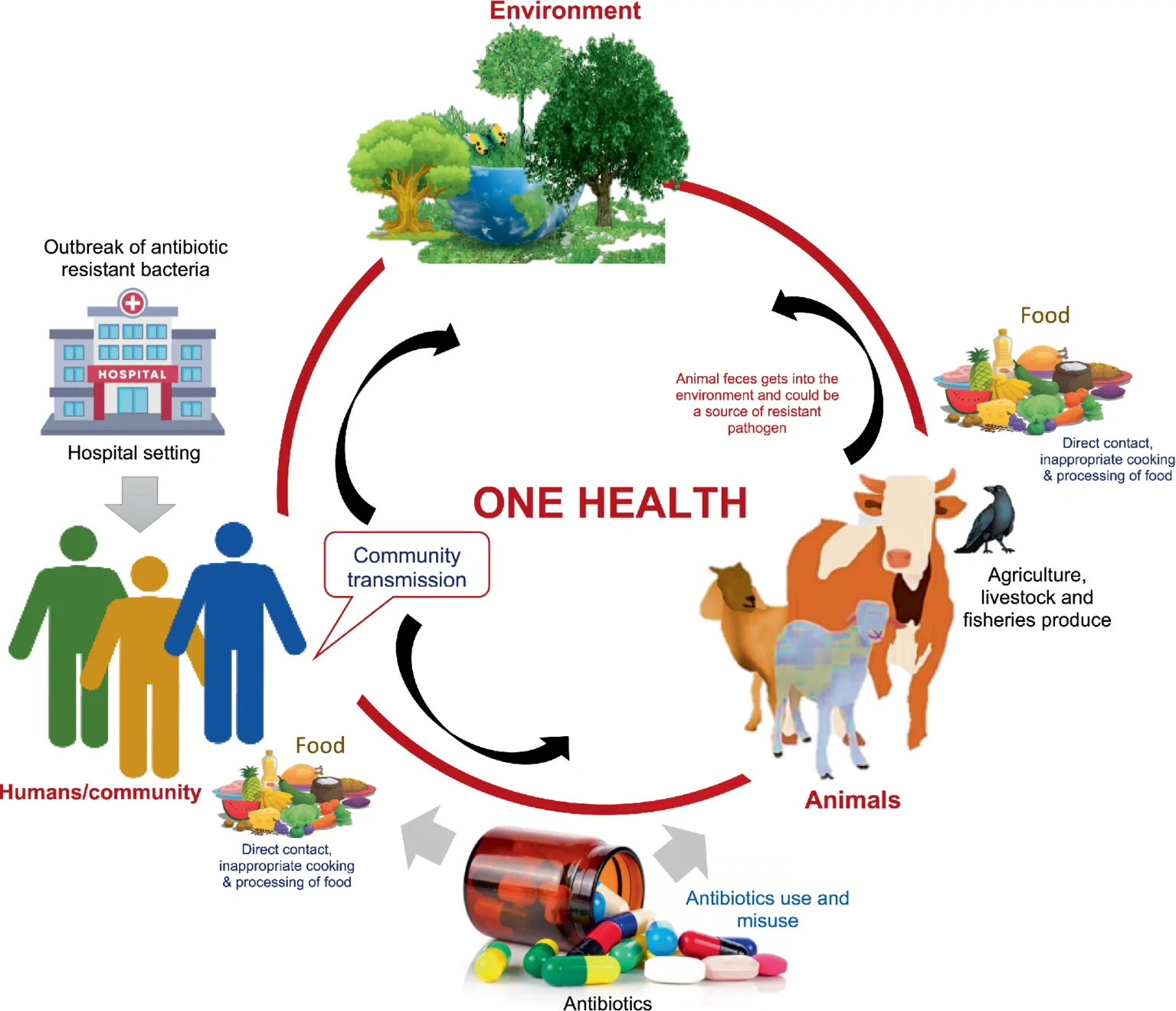
One Health pathways driving the emergence and transmission of antimicrobial resistance. This figure illustrates the interconnected transmission of antimicrobial resistance (AMR) across human, animal, food, and environmental systems within a One Health framework. Bidirectional pathways show how antibiotic use in healthcare, livestock production, and aquaculture selects for resistant bacteria that spread through food systems, direct contact, wastewater, and environmental contamination. Hospitals and communities act as major transmission hubs, while environmental reservoirs facilitate persistence and amplification of resistance. The figure highlights the continuous exchange of resistant bacteria and resistance genes across ecological boundaries, emphasizing the need for coordinated One Health interventions integrating human, animal, and environmental health sectors.

The One Health approach to AMR recognizes that resistance emerges and spreads through interconnected selective pressures across human, animal, and environmental sectors, making the problem complex but also offering opportunities for integrated solutions (17, 18). This framework highlights the cyclical and interconnected nature of AMR dissemination at the interfaces of these domains, emphasizing that resistant bacteria genes move between humans, animals, and the environment due to factors like antimicrobial misuse, pollution, and migration (19, 20). Effective One Health strategies involve coordinated actions such as improving antimicrobial stewardship in healthcare and agriculture, enhancing infection control, regulating antimicrobial use, and reducing environmental contamination (17, 21). Surveillance systems adopting a One Health perspective integrate data from multiple sectors to generate actionable knowledge that informs policy and intervention decisions (22, 23). Recent advances include global initiatives like the WHO Global Action Plan on AMR and collaborative global efforts to promote sustainable interventions across sectors (19, 24). Overall, this conceptual framework supports multisectoral collaboration to preserve antimicrobial efficacy and mitigate AMR’s impact on health, food security, and ecosystems worldwide (25, 26). Figure 1 emphasizes the interconnected and cyclical nature of AMR dissemination across human, animal, and environmental interfaces.

**Figure 2:**
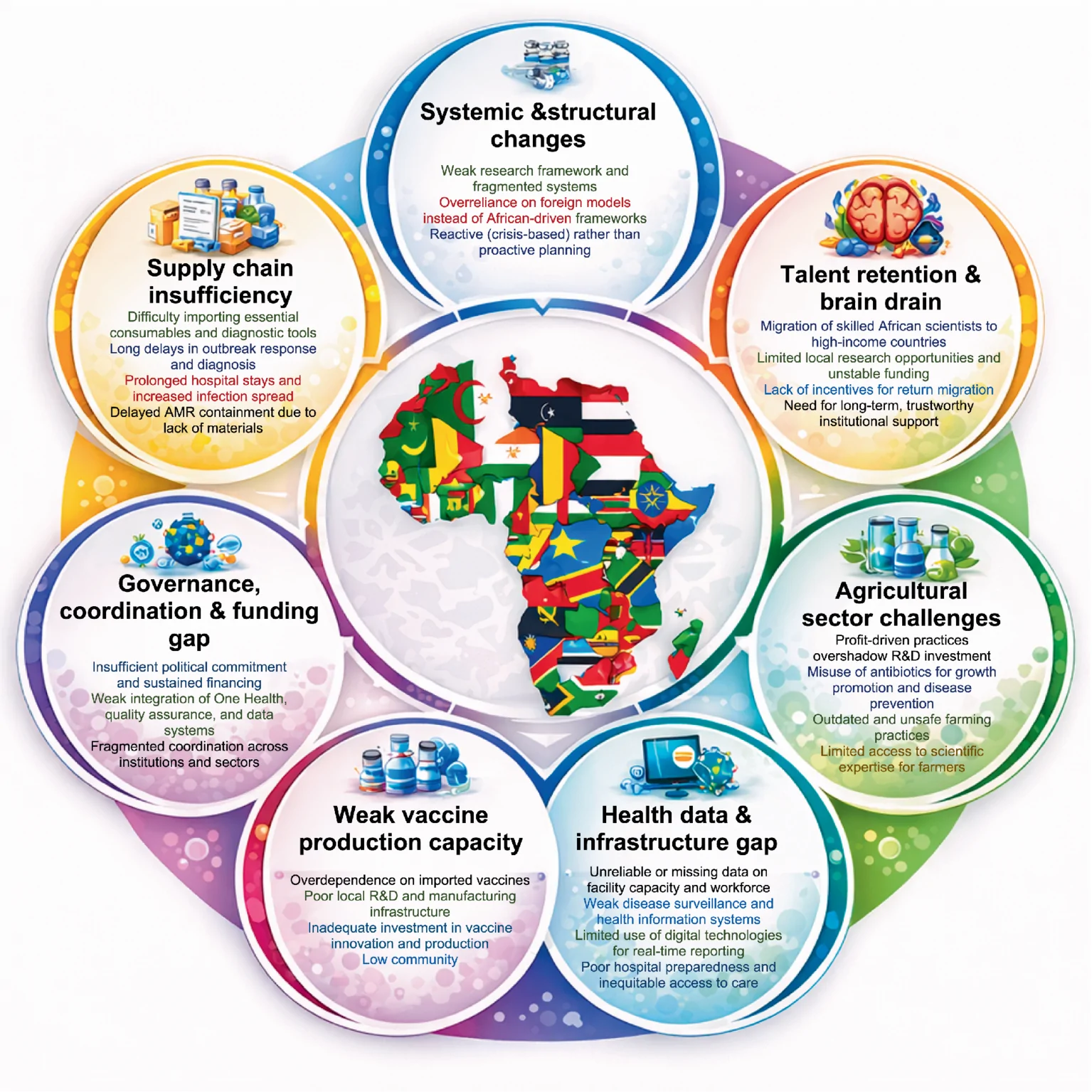
Challenges to curbing AMR in Africa. This figure illustrates the interconnected systemic barriers undermining effective One Health implementation and AMR preparedness in Africa. Centered on the continent, it highlights key, interrelated challenges across governance and financing, supply chain systems, health data and infrastructure, genomic and pandemic preparedness, vaccine production capacity, agricultural practices, talent retention, and broader structural research ecosystems. Together, these gaps constrain surveillance, response, innovation, and sustainability, reinforcing AMR transmission across human, animal, and environmental interfaces and underscoring the need for coordinated, Africa-driven, multisectoral solutions.

One Health strategies advocate for multisectoral collaboration, integrating surveillance, stewardship, and regulatory measures to address antibiotic use in healthcare, agriculture, and environmental management, as seen in national action plans and regional initiatives like those in Southeast Asia and South Korea (24, 27, 28). African LMICs have made notable progress in adopting the One Health approach, which integrates human, animal, and environmental health sectors. However, persistent implementation gaps remain, including weak governance and coordination structures, poor data sharing, limited integrated surveillance systems, and inadequate sustainable financing, with heavy reliance on external donor funding rather than government institutionalization (29, 30). Challenges also include limited political will, workforce capacity deficits, and fragmented multisectoral collaboration, which hinder effective One Health operationalization across many African countries (31–33) (Figure 2). Despite institutional momentum and functional coordination mechanisms, planning, learning systems, and intersectoral trust-building require strengthening to improve outcomes and scalability (34).

**Figure 3:**
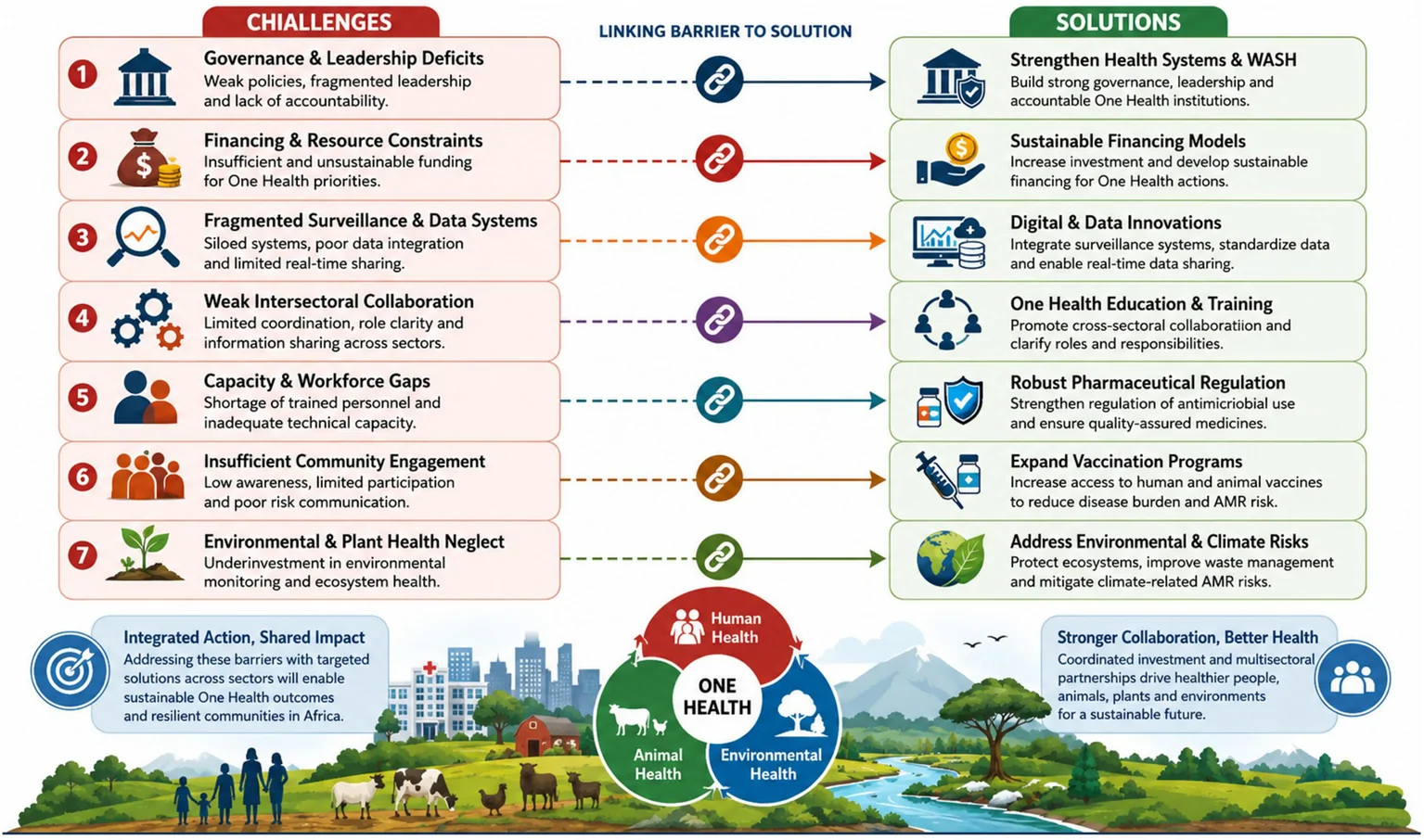
Barriers to One Health implementation in Africa and solutions.

So far several countries in Africa have made substantial progress in utilizing One health to addressing AMR despite several challenges to its implementations (Figure 3) and lessons have been learned from their approach. These lessons will be highlighted later in this review. As discussed in the following section, advances in genomic surveillance technologies have enhanced the ability to track AMR genes and pathogens across interconnected reservoirs, enabling source attribution and modeling of AMR transmission, as well as elucidating the role that mobile genetic elements play in disseminating resistance and virulence genes across One Health systems (35, 36). Overall, a coordinated, interdisciplinary One Health framework is essential to slow AMR dissemination, improve surveillance, and develop effective mitigation policies worldwide (24, 35).

## Mobilome and mobile genetic elements in AMR dissemination

### Plasmid-mediated dissemination across one health interfaces

Plasmids and associated MGEs form an interconnected “mobilome” that drives the movement of AMR genes between humans, animals, and environmental reservoirs (37). Broad-host-range plasmid taxonomic units, including IncP, IncF, IncI, IncX, and IncHI plasmids, are particularly important because they can colonize diverse bacterial hosts across ecological niches, thereby serving as major conduits for AMR genes dissemination (38, 39). These plasmids frequently accumulate multidrug resistance regions and interact with other MGEs, enabling rapid reshaping of resistance gene content under selective pressure (40).

Several studies now demonstrate extensive plasmid sharing across One Health sectors. In France, livestock- and companion animal-associated *Staphylococcus aureus* carried mosaic and hybrid plasmids that moved repeatedly between animal hosts, illustrating ongoing interspecies assembly of resistance determinants (41). Similarly, IncX1 plasmids carrying tet(X4) have been identified in clinical, livestock, and farm-environment *Escherichia coli,* supporting the existence of cross-sectorial plasmid transmission networks (42). Plasmid-mediated dissemination is also central to the global spread of mcr-1, which is predominantly associated with IncI2, IncHI2, IncX4, and IncFIB plasmids circulating across clinical, agricultural, food-chain, and environmental settings (43).

**Figure 4:**
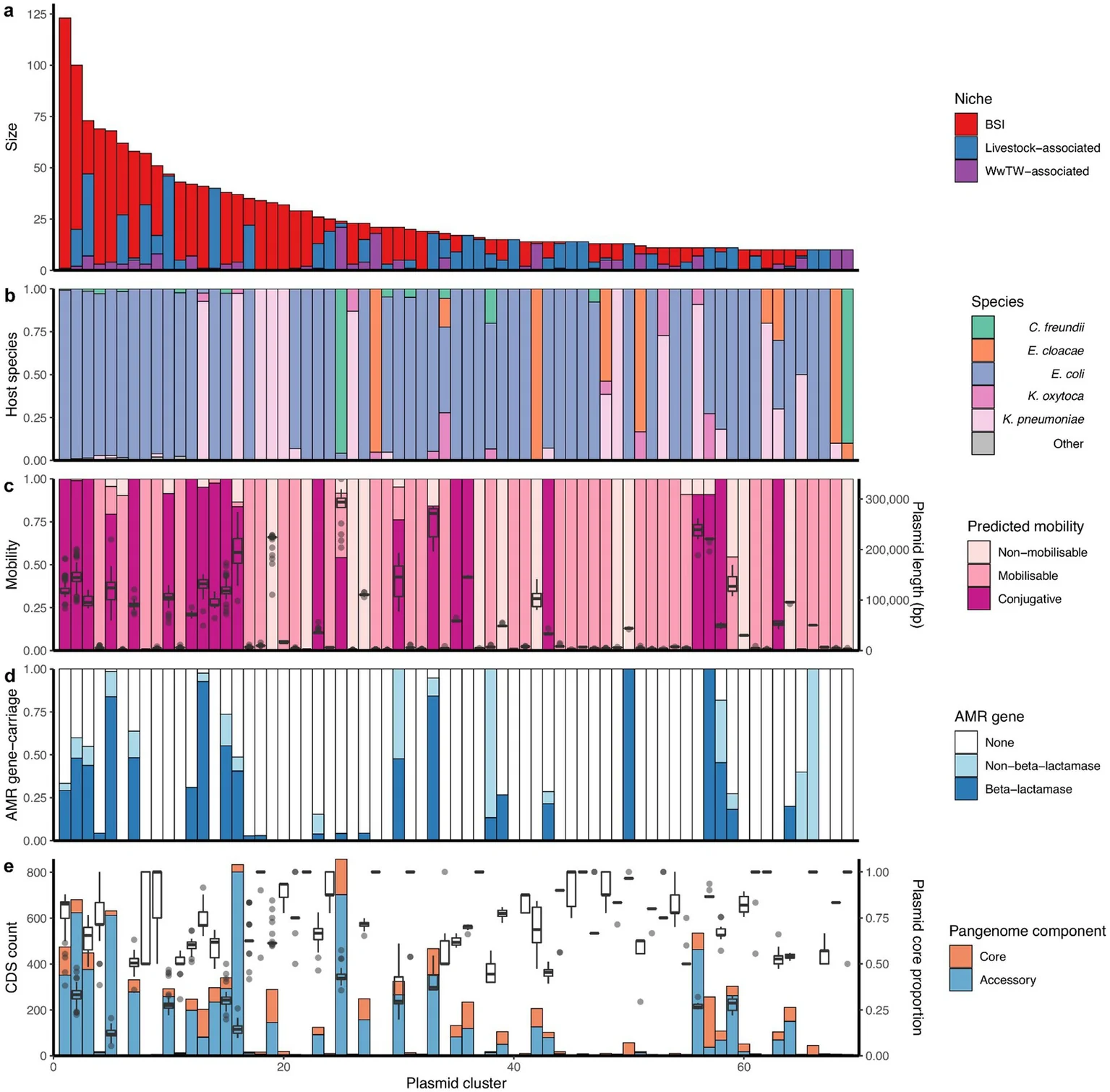
Ecological distribution, host range, mobility, and antimicrobial resistance profiles of major plasmid clusters across One Health reservoirs. Complete plasmid assemblies from *Enterobacterales* isolated from human bloodstream infections, livestock, farm soils, wastewater, and waterways in Oxfordshire, UK (2008–2020) were clustered based on shared backbone and pangenome similarity. **(a)** Distribution and size of plasmid clusters across reservoirs. **(b)** Broad host range of major plasmid clusters among *Escherichia coli*, *Klebsiella pneumoniae*, *Enterobacter cloacae*, *Klebsiella oxytoca*, and *Citrobacter freundii*. **(c)** Relationship between plasmid size and predicted mobility, showing that larger plasmids are predominantly conjugative or mobilisable. **(d)** Distribution of antimicrobial resistance genes across plasmid clusters, highlighting enrichment of clinically relevant resistance determinants. **(e)** Association between plasmid size and gene content, with larger plasmids exhibiting greater accessory gene diversity and genomic plasticity. Adapted from Matlock et al. (44).

Large-scale genomic surveys further reinforce the role of plasmids as ecological bridges linking distinct reservoirs. In Oxfordshire, highly similar Enterobacterales plasmid backbones were recovered from human bloodstream infections, livestock feces, wastewater, and farm soils, indicating frequent cross-interface transfer followed by local adaptation of accessory regions (44) (Figure 4). Collectively, these findings demonstrate that conjugative plasmids function as dynamic reservoirs that sustain AMR circulation across interconnected One Health systems.

### Integrons, transposons, and insertion sequences as drivers of genetic plasticity

While plasmids mediate intercellular transfer, integrons, transposons, and insertion sequences (ISs) facilitate the capture, rearrangement, and mobilization of AMR genes within and between plasmids and chromosomes. Integrons provide site-specific recombination platforms that capture resistance gene cassettes, whereas transposons and IS elements enable their movement across diverse genomic contexts (12, 45). Class 1 integrons are particularly important because they are frequently embedded within conjugative plasmids and transposons, creating highly connected networks of horizontal gene transfer. Increasing genomic evidence suggests that IS elements, especially IS26 and members of the IS6 family, play central roles in plasmid evolution by reshaping multidrug resistance regions and promoting inter-plasmid exchange of clinically important AMR genes (46). IS26 has been strongly linked to the dissemination of carbapenemase genes such as *bla*_NDM-1_ across bacterial species and hospital outbreaks through rapid restructuring of composite transposons and resistance islands (47).

Similarly, IS6-family elements facilitate movement of the oxazolidinone resistance gene *optrA* among mobilizable and conjugative plasmids, enhancing dissemination between manure-associated Enterococcus populations and human-associated strains (48). For colistin resistance, ISApl1-mediated composite transposons such as Tn6330 have enabled widespread mobilization of *mcr-1* between chromosomes and plasmids in isolates recovered from livestock, wastewater, food products, and clinical samples (43). Together, these elements provide the genomic flexibility required for rapid assembly and redistribution of multidrug resistance determinants across bacterial populations.

### Co-selection mechanisms and environmental drivers of mobilome expansion

Beyond antibiotic exposure, environmental co-selective pressures strongly influence mobilome dynamics and persistence. AMR genes frequently co-occur with metal resistance genes (MRGs) and biocide resistance determinants on the same plasmids or chromosomal MGEs, allowing non-antibiotic contaminants to indirectly maintain antimicrobial resistance within bacterial communities (45, 49). Broad-host-range plasmids, particularly IncP plasmids, commonly carry linked AMR genes–MRG regions that remain selectively advantageous in environments contaminated with heavy metals such as mercury, copper, zinc, chromium, and silver (46). Experimental studies further demonstrate that environmentally relevant concentrations of these metals can directly enhance conjugative plasmid transfer by inducing oxidative stress responses, increasing membrane permeability, and upregulating conjugation-associated genes (50). These findings suggest that environmental pollution can actively accelerate horizontal gene transfer independently of antibiotic exposure.

Hospital wastewater and agricultural runoff represent important co-selection hotspots where AMR genes, MRGs, disinfectant resistance genes, and MGEs converge. Metagenomic analyses of hospital wastewater have shown extensive co-occurrence of plasmid-associated AMR genes with mercury resistance operons and qacEΔ1-mediated disinfectant resistance genes in high-risk pathogens such as *Klebsiella* and *Enterococcus* (51). However, despite strong evidence for co-occurrence patterns, several proposed co-selection relationships remain largely correlational and require functional validation under environmentally relevant conditions.

### Ecological connectivity and cross-reservoir transmission

The mobilome operates within highly interconnected ecological systems rather than isolated bacterial populations. Antimicrobial use in livestock selects for resistant bacteria carrying conjugative plasmids and transposable elements, which are subsequently released into soils, wastewater, and aquatic systems where AMR genes can spread horizontally to environmental bacteria (52, 53). Environmental reservoirs then act as amplification points from which resistant organisms may re-enter human populations through contaminated food, water, wildlife, or direct contact. Hospital wastewater represents a particularly important transmission interface because it contains dense mixtures of clinical pathogens, antibiotics, disinfectants, and mobile resistance elements that facilitate ongoing plasmid exchange between environmental and healthcare-associated bacteria (54, 55). Wildlife additionally contributes to long-distance dissemination of resistant bacteria across urban and natural ecosystems, linking otherwise disconnected reservoirs (56).

Overall, current evidence supports a model in which plasmids, integrons, transposons, and insertion sequences collectively sustain a highly dynamic mobilome that continuously redistributes AMR genes across human, animal, and environmental interfaces. Addressing AMR therefore requires integrated One Health interventions that extend beyond antimicrobial stewardship alone to include wastewater management, environmental monitoring, agricultural regulation, and genomic surveillance of mobile resistance elements.

### The animal–environment interface as a core reservoir of AMR genes

Research on AMR in Africa highlights the animal–environment interface as a significant reservoir and transmission point for AMR genes, though data remain limited. Genomic epidemiology in Nairobi revealed that AMR gene diversity is shared across humans, livestock, and wildlife, with environmental factors like poor manure disposal increasing transmission risk at the human-livestock interface (57). Wildlife studies from Senegal and East Africa demonstrate that wild animals harbor diverse antimicrobial resistance genes and resistant bacteria, even in settings with limited direct antibiotic exposure, highlighting their potential roles as both reservoirs and sentinels of environmental AMR dissemination (58, 59). Similar findings have been reported across Europe, the Americas, and Antarctica, suggesting that wildlife-associated AMR is a globally distributed phenomenon rather than one restricted to regions with intensive antimicrobial use. Across these settings, resistant *Escherichia coli*, extended-spectrum *β*-lactamase (ESBL)-producing bacteria, and clinically important resistance genes have been detected in diverse wildlife species, including birds, mammals, and reptiles (60–62). A notable area of agreement across studies is the consistent detection of AMR in wildlife inhabiting both human-impacted and relatively remote environments. This pattern supports the hypothesis that wildlife primarily acquires resistant bacteria and genes through environmental contamination pathways, including wastewater discharge, agricultural runoff, and interactions with livestock or human-associated ecosystems. However, studies differ in the extent to which anthropogenic exposure predicts AMR prevalence. While some investigations report higher resistance levels in wildlife populations located near human settlements or agricultural activities, others have detected substantial AMR burdens even in remote populations, indicating that AMR dissemination is influenced by complex ecological and environmental processes rather than simple proximity to human activity alone (63–65).

Collectively, the evidence supports a dual role for wildlife within the One Health framework: wildlife can act as reservoirs that maintain and disseminate resistant organisms and genes across ecological boundaries, while simultaneously serving as sentinels that reflect the extent of environmental AMR contamination (56, 66). Nevertheless, the strength of this evidence is constrained by important limitations, including small sample sizes, heterogeneous methodologies, limited longitudinal surveillance, and challenges in determining the directionality of AMR transmission between wildlife, humans, livestock, and the environment. These unresolved questions underscore the need for standardized surveillance approaches and integrated genomic investigations that can better elucidate wildlife’s contribution to mobilome-mediated AMR dissemination. Several evidence are available showing that wildlife act not only as passive carriers but as ecological bridges through which plasmid-mediated resistance can circulate between natural and human-impacted systems (67–70).

Studies from Nigeria demonstrate substantial MDR among *E. coli* isolates recovered from poultry, farm workers, and farm-associated environments, with evidence of genetic relatedness across human, animal, and environmental sources, suggesting transmission along the food production chain (71, 72). Similar patterns have been reported globally, where MDR and ESBL-producing *E. coli* are frequently detected in food animals, individuals with occupational exposure to livestock, and surrounding environments. Studies from Bangladesh, Egypt, Europe, and Latin America consistently identify poultry production systems as important interfaces for the circulation of resistant bacteria and resistance genes between humans, animals, and the environment (73–76).

A major area of agreement across these studies is the central role of MGEs, particularly plasmids, insertion sequences, and transposons, in facilitating the dissemination of antimicrobial resistance genes across ecological boundaries. In Nigeria, multidrug-resistant *E. coli* carrying MGEs that encode both resistance and virulence determinants provide evidence for ongoing genetic exchange among bacterial populations from different hosts (77). Comparable observations have been reported in Asia and Europe, where resistance genes are frequently associated with epidemic plasmids capable of moving between bacterial strains, host species, and environmental reservoirs (78, 79). These findings suggest that mobilome-mediated dissemination is a common mechanism underpinning AMR spread across geographically distinct settings. However, the dominant mechanism of dissemination may vary according to local production systems, environmental conditions, and antimicrobial use practices (71, 72, 75, 76, 78, 79).

Another consistent theme across regions is the importance of environmental reservoirs. While Nigerian studies included farm environments as part of the transmission network, research from Bangladesh, Slovenia, Ecuador, and India further demonstrates that wastewater, rivers, soil, market environments, and animal waste can serve as important reservoirs and conduits for resistant bacteria and mobile resistance elements (75, 76, 78, 80). Collectively, these findings support a One Health model in which AMR dissemination is driven by interconnected transmission pathways linking humans, animals, and environmental compartments. Taken together, the Nigerian evidence aligns with a broader global pattern characterized by widespread MDR and ESBL-producing *E. coli*, extensive sharing of resistance determinants through MGEs and transmission occurring through both food-chain and environmental routes. Although the relative contributions of clonal spread and horizontal gene transfer remain incompletely resolved, the available evidence consistently highlights the need for integrated genomic surveillance that simultaneously incorporates human, animal, and environmental sampling. Such approaches are essential for identifying dominant transmission pathways, tracking high-risk resistance plasmids and clones, and informing effective One Health interventions to mitigate AMR dissemination.

Although several studies have identified pets, particularly companion animals such as dogs and cats, as important reservoirs and potential transmission sources of AMR and MDR pathogens (81–83), the evidence remains inconsistent. For example, Cuevas-Espelid et al. (84) reported no direct evidence of MDR bacterial transmission between healthy pets and their owners, highlighting ongoing uncertainty regarding the frequency and epidemiological significance of interspecies transmission in household settings. These contrasting findings may reflect differences in study design, sampling strategies, geographical settings, bacterial species investigated, and the use of genomic approaches to infer transmission. Collectively, the current evidence suggests that while pets may serve as reservoirs of resistant bacteria, the extent to which they contribute to sustained human transmission remains incompletely understood. This gap is particularly evident in African settings, where longitudinal surveillance studies and high-resolution genomic epidemiology data are still limited.

Wastewater treatment plants (WWTPs) are also recognized as critical hotspots for the dissemination of AMR (85). While conventional treatment processes can reduce ARB, they often fail to effectively remove AMR genes, leading to their release into natural water bodies and contributing to environmental AMR spread (86–88). Surface waters and aquatic products have been shown to harbor plasmids carrying resistance genes against quinolones, tigecycline, colistin (mcr-1), and other antibiotics, highlighting aquatic ecosystems as reservoirs and conduits for multidrug resistance dissemination (89, 90). The diversity of plasmids and AMR genes in aquatic bacteria, along with their association with mobile genetic elements, underscores the complexity of resistance gene flow and the role of environmental factors in accelerating antimicrobial resistance spread (89, 91). Clonal spread of pandemic MDR *E. coli* lineages such as ST131 across humans, animals, wildlife, and wastewater has also been reported (92). Carbapenem-resistant *Acinetobacter baumannii*, particularly strains harboring the *bla*_OXA-23_ gene, are also commonly found in hospital sewage and soil environments, serving as reservoirs that contribute to the persistence and spread of this pathogen (93–96). MDR *Pseudomonas aeruginosa* resistant to carbapenems and fluoroquinolones is frequently found in hospital plumbing, water, and soil environments, where it forms biofilms that enhance persistence and resistance (97–99). Multiple outbreaks linked to contaminated sinks, drains, and faucets, often involving high-risk clones such as ST111 and ST235 carrying carbapenemase genes like VIM-2 has also been reported (100–102). Environmental isolates from community water sources and soil also show high rates of carbapenem resistance and MDR (81, 103–106).

Airborne transmission is also increasingly recognized as a significant but neglected route for spreading AMR and MDR pathogens. Urban ambient air in megacities harbors site-specific AMR genes linked to pathogenic taxa, with hospital air enriched in resistance to rifamycin and glycopeptides, highlighting persistent airborne antibiotic resistance hazards (107). Additionally, bioaerosols in coastal and estuarine environments contain opportunistic pathogens like *Burkholderia* and *Acinetobacter* alongside diverse AMR genes, emphasizing the need to include airborne resistomes in environmental and health risk assessments (108, 109). Studies highlight that horizontal gene transfer (HGT) in the atmosphere further promotes the spread of AMR, emphasizing the need for biomonitoring airborne AMR genes to inform health advisories (110). Airborne AMR genes and MDR pathogens, including methicillin-resistant *Staphylococcus* spp., have been detected up to several kilometers from chicken and dairy farms, with over 80% of airborne *Staphylococcus i*solates from poultry environments carrying the *mecA* gene and multiple resistance genes (111, 112). Swine farms release airborne bacteria and AMR genes mainly conferring resistance to tetracyclines, aminoglycosides, and lincosamides, with swine feces contributing substantially to the airborne resistome; inhalation exposure to these pathogens by farm workers is orders of magnitude higher than in hospital environments (113, 114). Residential areas near livestock farms also show detectable levels of livestock-associated bacteria and AMR genes such as *tetW* and *mecA*, with concentrations correlating to farm density, especially poultry and pig farms (115, 116). The detection of AMR genes in bioaerosols further suggests that MGEs contribute to the airborne dissemination of resistance, as plasmid- and transposon-associated genes can be transferred between bacteria in aerosolized particles, extending the spatial range of horizontal gene transfer beyond direct environmental contact.

Overall, this study highlights that AMR across animal and environmental reservoirs is not solely driven by the presence of resistant bacteria, but by the dynamic activity of MGEs that mediate gene exchange across ecological boundaries. Plasmids, integrons, and transposons facilitate the movement of AMR genes between livestock, wildlife, companion animals, and environmental compartments such as water, soil, and air. These MGEs transform discrete reservoirs into a highly interconnected network, where AMR genes can be continuously reshuffled, amplified, and transmitted between sectors. Consequently, animals and environmental matrices should not be viewed merely as passive recipients of human-derived AMR, but as active participants in a feedback loop sustained by HGT. This reinforces the importance of a One Health framework that explicitly considers the role of MGEs in linking AMR across human, animal, and environmental systems.

**Figure 5:**
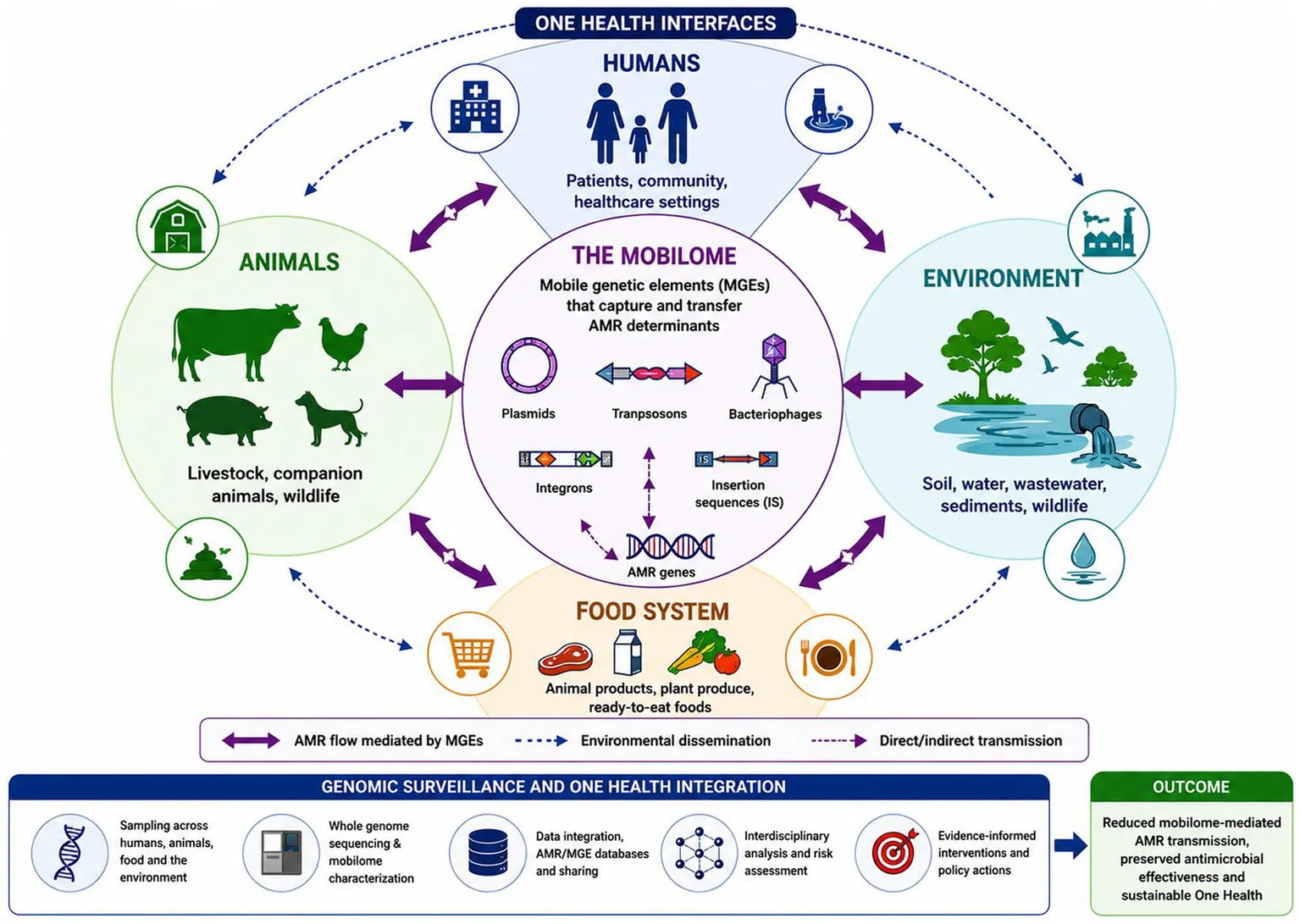
Conceptual framework illustrating mobilome-driven dissemination of antimicrobial resistance across One Health interfaces. This figure illustrates the central role of the bacterial mobilome in the dissemination of AMR across interconnected One Health interfaces, including humans, animals, food systems, and the environment. MGEs, such as plasmids, transposons, integrons, IS elements, and bacteriophages, facilitate the capture, mobilization, and horizontal transfer of AMR determinants between bacterial populations across diverse ecological niches. Bidirectional transmission pathways occur through direct and indirect contact, food production systems, environmental contamination, wastewater discharge, and wildlife-associated dissemination. The framework also highlights major drivers of mobilome activity and AMR spread, including antimicrobial misuse in human and veterinary medicine, agricultural intensification, industrial and environmental contamination, globalization, and climate-related ecological disturbances. Enabling factors such as inadequate water, sanitation, and hygiene (WASH) infrastructure, poor waste management, close human–animal interactions, and limited genomic surveillance capacity further accelerate AMR dissemination, particularly in many African settings. Integrated genomic surveillance within a One Health framework is essential for tracking mobilome-associated resistance transmission, identifying emerging high-risk clonal lineages and MGEs, and informing evidence-based intervention.

Figure 5 summarizes the conceptual framework illustrating mobilome-driven dissemination of antimicrobial resistance across One Health interfaces.

### Challenges and Africa-specific strategies for integrated one health genomic surveillance

Despite growing recognition of AMR as a critical One Health challenge, Africa continues to face substantial barriers to effective genomic surveillance of AMR and its associated MGEs across human, animal, and environmental health systems (117, 118). A major limitation is the inadequate laboratory and sequencing infrastructure in many African countries. Genomic tools such as whole-genome sequencing, metagenomics, and plasmid-resolved analyses are largely restricted to a small number of reference laboratories and academic centers, leading to uneven surveillance coverage and reliance on external facilities for sequencing and bioinformatics (10, 35, 119, 120). This constrained capacity limits routine detection and tracking of plasmids, transposons, integrons, and other MGEs that drive resistance gene dissemination across diverse bacterial populations and ecosystems (121–123).

Another major challenge is the limited integration of One Health surveillance systems. In many settings, AMR monitoring remains fragmented, with human clinical surveillance operating separately from veterinary, agricultural, and environmental programs, despite calls for integrated AMR data streams and systems-thinking approaches (118, 124–126). This siloed architecture hampers the ability to trace the movement of resistance genes and MGEs across interconnected reservoirs such as hospitals, livestock farms, wastewater systems, and surface waters, even though these compartments are recognized as key AMR interfaces (8, 127–129). Because MGEs frequently mediate HGT across these interfaces, the lack of coordinated, cross-sectoral data integration substantially weakens epidemiological inference and the design of effective interventions (25, 35).

Africa also faces significant resource and funding constraints that limit the development and sustainability of genomic surveillance programs. Many AMR and genomics initiatives are short-term, project-based, and heavily reliant on external donors, resulting in episodic data generation without long-term surveillance continuity (130–133). The high costs of sequencing platforms, reagents, maintenance, computing infrastructure, data storage, and skilled personnel remain major obstacles, particularly in low-resource settings (119, 133–135).

Limited bioinformatics expertise and data-management capacity further constrain impact. Effective mobilome analysis requires advanced computational tools for plasmid reconstruction, resistome profiling, and comparative genomics, but many laboratories lack sufficient high-performance computing and robust data systems (120, 136). Persistent shortages of trained genomic epidemiologists and bioinformaticians across the continent hinder routine analysis of sequencing data and delay its translation into timely public health action (135–139).

A further Africa-specific concern is the widespread unregulated use of antimicrobials in both human and animal health sectors. In many settings, antibiotics are easily accessible without prescription, and antimicrobial use in livestock production is often poorly regulated and rarely captured in formal surveillance systems (130, 140, 141). High levels of antimicrobial use in food-producing animals and weak stewardship create strong selective pressures that promote the persistence and spread of resistance genes on transferable MGEs in clinical, community, and environmental reservoirs (8, 130).

Addressing these challenges requires continent-specific, sustainable solutions. Priority actions include investment in regional sequencing hubs and shared computing infrastructure; harmonized One Health surveillance frameworks; cross-border, Africa-owned data-sharing platforms; and large-scale workforce development in genomics, bioinformatics, and genomic epidemiology (133, 136, 139). Strengthening antimicrobial stewardship policies in human and animal health, alongside expanding Africa-led collaborative networks such as H3Africa, Africa PGI, H3ABioNet, and regional training initiatives, will be essential for capturing the dynamics of the mobilome and informing targeted AMR control strategies.

### Major recurring mobilome–AMR patterns at African one health interfaces

Across African One Health studies, HGT via MGEs is consistently identified as the dominant mechanism moving resistance determinants within and between bacterial populations. MGEs capture and recombine AMR genes, enabling rapid emergence of MDR and extensively drug-resistant pathogens across environments and hosts (121, 142, 143). Co-selective pressures from antimicrobials, heavy metals and other pollutants in clinical, agricultural and environmental settings further amplify these MGE-mediated processes (144, 145).

For recurring MGEs and resistance gene contexts, African carbapenemase and Enterobacterales studies consistently report specific plasmid backbones and accessory MGEs as central hubs for AMR genes. Distinct plasmid replicons, integrons, transposons and insertion sequences flank carbapenemase genes across human, animal and environmental isolates, supporting intra- and inter-species transmission across sectors (146, 147). In South African CRE, IncFIB(K), IncL/M(pOXA48-like) and Col-type plasmids, together with class 1 integrons, IS elements and Tn3-like transposons, anchor and co-mobilize carbapenemase and extended *β*-lactamase genes between human and environmental reservoirs within the same geography (146, 147). In One Health *E. coli* scoping work, plasmids, integrons, insertion sequences and transposons are likewise repeatedly linked to ESBLs, colistin resistant gene and other AMR genes across human, animal and environmental isolates, underscoring a shared mobilome architecture at African interfaces (148).

As for major transmission pathways across One Health interfaces, evidence from African and global One Health studies converges on animal–environment and food-chain routes as key conduits for mobilome-driven AMR dissemination. In African drug resistant *E. coli*, the food chain is the most frequently inferred transmission route linking humans, animals and environmental compartments (148). South African CRE work shows near-identical plasmid–MGE–AMR gene constellations in human and surface-water isolates from the same area, indicating environmental waters as bridging reservoirs for carbapenemase genes between community and clinical settings (147). More broadly, polluted water, soil and air are highlighted as dynamic environmental reservoirs where MGEs, biofilms and low-level pollutants promote AMR gene exchange and persistence, further connecting human, livestock and wildlife microbiomes (144, 149).

Despite increasing initiatives, AMR genomic surveillance in Africa remains fragmented and under-resourced, especially for systematic mobilome tracking across One Health sectors. Only a minority of African countries had fully implemented AMR surveillance systems by 2019, and existing systems are mostly uncoordinated, leaving significant data gaps on resistance patterns and transmission (131, 150). WGS and gene/plasmid-centric approaches can resolve mobilization events and cross-sector transmission more clearly than isolate-only analyses, but access, standardization, and integration into routine clinical microbiology remain limited across much of the continent (35, 131, 151). Even in focused One Health *E. coli* work, contemporary AMR data are missing for over two-thirds of African countries, underscoring substantial geographic blind spots for mobilome–AMR mapping (148).

In summary, African One Health studies reveal a recurrent pattern in which a shared mobilome – dominated by specific plasmid families, integrons, insertion sequences and transposons – moves key AMR genes along food-chain and environmental pathways linking humans, animals and ecosystems. The food chain continue to emerges as the most inferred transmission route of antibiotic-resistant bacteria in African settings (148), with animal-environmental interfaces serving as critical hotspots for mobilome-driven resistance emergence. However, significant surveillance gaps persist: environmental AMR monitoring remains fragmented and underdeveloped (127), and integrated One Health studies simultaneously examining human, animal, and environmental compartments remain scarce across Africa. These gaps fundamentally limit understanding of mobilome dynamics and cross-sectoral transmission pathways essential for effective AMR control. Table 1 summarizes genomic surveillance studies on AMR mobilome in a One Health context.

**Table 1:** African genomic surveillance studies on AMR mobilome in a One Health context.

Country	Sample source(s)	Bacterial species	AMR genes detected (key examples)	Mobile genetic elements identified	Sequencing/molecular method used	Main surveillance implications	Citations
South Africa	Pig farm, abattoir, transport, pork products	*Escherichia coli*	blaEC, blaTEM, blaAmpC, tetA/B/34, sul1/2, aadA1	24 plasmid replicons, 43 prophages, 19 IS families, 7 class 1 integrons	WGS (Illumina)	High diversity of plasmid-borne AMR genes and MGEs across farm-to-fork; need for routine genomic surveillance in livestock systems	Chekole et al. (165)
Nigeria	Rectal swabs from pigs and poultry layers	*Escherichia coli*	qnrS1/10/15, kdpE, cmlA1, MIR-14, sul3, dfrA12	Plasmids (various), MGEs linked to detected AMR genes	WGS (Nanopore)	Rapid field-deployable sequencing feasible; highlights need for robust pipelines and animal sector surveillance	Richter et al. (166)
Ghana	Urban surface water, hospital wastewater	*Escherichia coli*	TEM-1B/C, CTX-M-15, blaDHA-1, OXA-1/181	Col440I plasmid (10.2%), IncFIB(AP001918) (6.8%)	WGS (Illumina)	Urban water is a reservoir for MDR *E. coli*; environmental monitoring critical for public health	Hurtado et al. (167)
Kenya	Clinical isolates from hospitals	*Klebsiella pneumoniae*	blaNDM-1, blaOXA-181, armA/rmtF (aminoglycoside), CTX-M-15/TEM/OXA	Plasmids: mcr-8 detected; various others	WGS (Illumina)	Diverse resistome and virulome; high-risk clones with carbapenemase genes present in community and hospital	Hossein and Ripanda (168)
Ethiopia	Calves, humans, environmental samples	*Escherichia coli*	bla-ampH/AmpC1 (90%+), tet(A), mdf(A), aph(3″)-Ib/6-Id/sul2/bla-PBP	Tn3 with bla-TEM-105; Int1 with sul1/dhfr7	WGS (Ilumina)	Extensive AMR genes/MGE distribution across sources; horizontal gene transfer likely driver	Hassen et al. (169)
Ghana	Sheep feces & river water	*Klebsiella pneumoniae/variicola/michiganensis*	fosA6/LEN24/OXY-1-3/tet(34)/aph(3′)-Ia/mdf(A)/oqxAB	AB595 plasmid in *K. pneumoniae*; other plasmids	WGS (illumina)	Rural environments are reservoirs for MDR *Klebsiella* spp.; need for integrated One Health surveillance	Viviers et al. (170)
Malawi/Uganda	Stool (human & animal), hand-contact surfaces, food/water samples	*Escherichia coli* (ESBL-producing)	blaCTX-M-15 (68%), blaCTX-M-27 (14%)	55 + different plasmids carrying ESBL genes	WGS (illumina) + SNP/network analysis	Frequent spillover between compartments; complex plasmid networks drive dissemination	Olaitan et al. (171)
Nigeria	Recreational beach water & wet sand	Enterobacterales incl. *E. coli*	Multiple beta-lactamases incl. blaNDM-1	Plasmids/insertion sequences/transposons; ISKpn14 upstream of blaNDM-1	WGS (Illumina)	Beaches contaminated with clinically relevant AMR genes on MGEs – potential transmission at human–environment interface	Richter et al. (172)
South Africa	Irrigation water & fresh produce	*Escherichia coli*	ESBL genes: CTX-M/TEM/OXA	Associated with mobile genetic elements	WGS (illumina)	Fresh produce and irrigation water as reservoirs for MDR *E. coli* – need for ongoing environmental surveillance	Dushayeva (173)
South Africa	Pig feces, transport, abattoir, carcass/processing environments	MDR *E. coli*	blaEC, blaTEM, blaAmpC; tetA, tetB, tet34; sul, dfr, aminoglycoside and phenicol genes	24 plasmid replicons; class 1 integrons; 19 IS families; prophages	WGS (Illumina) plus phenotypic AST	Reveals rich mobilome (plasmids, integrons, ISs) along the pork chain; supports integrating WGS into routine livestock AMR surveillance and abattoir control points	Abdalla et al. (163)
South Africa	Sheep feces; river water	*K. pneumoniae, K. variicola, K. michiganensis*	fosA, oqxAB, blaLEN/OXY, aph(3′)-Ia, tet(34); in *K. pneumoniae* also blaSHV-194, fosA6, tet(A)	Plasmid AB595 carrying tetA, sul, aph(3′)-Ia in *K. pneumoniae*	WGS (illumina)	Demonstrates rural small-stock and surface waters as reservoirs of plasmid-borne MDR *Klebsiella*; argues for One Health surveillance in low-resource farming communities	Motlhaping et al. (174)
South Africa	WWTP final effluent; upstream and downstream river water	Diarrheagenic MDR *E. coli*	blaTEM-1B, blaCTX-M-14, blaCTX-M-55; mcr-9; multiple AMR genes to several classes	Class 1 integrons (In22, In54, In191, In369); ISs (e.g., IS1, IS3, IS91); transposons (Tn402/Tn21); prophages	WGS (Illumina)	Effluent and river carry pathogenic MDR *E. coli* with ESBL and mcr-9 on integrons/transposons, often clustering with clinical isolates; WWTPs and rivers are critical AMR–mobilome surveillance nodes	Mbanga et al. (175)
South Africa	Human stool; environmental water; pig samples (no CRE)	*K. pneumoniae, K. quasipneumoniae, E. hormaechei, Citrobacter freundii, Serratia marcescens*	Carbapenemases (e.g., blaOXA-181, other blaKPC/blaNDM-types), multiple β-lactamases	IncFIB(K), IncL/M(pOXA-48/pMU407), Col440I plasmids; class 1 integrons; multiple ISs (IS91, IS5075, IS30, IS3000, IS3, IS19, ISKpn19), Tn3	WGS (illumina)	Shows carbapenemase genes shared on plasmids and integrons between human and environmental Enterobacterales; highlights environmental–human MGE-mediated carbapenemase dissemination and need for One Health containment	Ramsamy et al. (147)
South Africa	Fecal samples from food-chain animals	*Enterococcus faecalis, E. faecium, E. durans*	ant(6)-Ia, aph(3′)-IIIa, sat4, spw; erm(B); dfrG; tet(L), tet(M)	Multiple plasmid replicons in *E. faecalis* and *E. faecium*	WGS (Illumina), MLST, plasmid typing	Identifies novel STs and multidrug-resistant enterococci with plasmid-borne AMR genes in livestock; provides genomic baseline for enterococcal mobilome surveillance in African food animals	El Zowalaty et al. (164)
Kenya (Nairobi)	Human, livestock, wildlife feces	Commensal*/*pathogenic *E. coli*	56 acquired genes + 13 mutations across nine classes; ESBLs including blaCTX-M-15	Many AMR genes co-located on putative MGEs, enabling multi-drug resistance in a single step	WGS (illumina)	Shows overlapping AMR gene communities across humans, livestock, wildlife, with frequent co-location of AMR genes; supports urban One Health surveillance focused on waste/manure management and shared environments	El Zowalaty et al.(57)
Tanzania (plus Kenya subset)	Humans, livestock, fish, environment	*Escherichia coli*	Widespread aminoglycoside genes (aac(3)-IId, aph(3″)-Ib, aph(6)-Id, aadA1, aadA5) and *β*-lactam genes (blaTEM-1, blaOXA-1, blaCTX-M-15) across all sources; quinolone resistance mutations (gyrA_S83L, gyrA_D87N, parC_S80I); tetracycline genes tet(A/B/D) in humans, livestock, fish	Plasmid types IncFIA, IncI1, IncFII extensively shared between humans and livestock	WGS (illumina)- genomes from publically available database	Demonstrates interconnected AMR gene and plasmid flow across human–animal–environment in an East African setting; supports need for integrated One Health genomic surveillance	Lyimo and Sonola (176)
Tanzania (context from same group)	Drinking water sources; surgical site infections; poultry (referenced Tanzanian studies feeding into above WGS dataset)	Mainly *Escherichia coli, mixed pathogens*	High prevalence of antibiotic-resistant *E. coli* in drinking water and multidrug-resistant surgical site pathogens (no specific gene list in excerpt)	Not detailed in excerpt	Phenotypic resistance testing; earlier molecular work feeding data to later WGS meta-analysis	Show Tanzania as a source country for environmental and clinical AMR *E. coli* feeding into regional One Health genomic analyses	Lyimo and Sonola (176)
Tanzania (plus Kenya subset)	Humans, livestock, fish, environment (One Health)	*Escherichia coli*	Re-analysis of 174 WGS datasets (subset of 244 above) focusing on AMR gene and plasmid distribution in Tanzanian isolates; similar gene profiles (aminoglycosides, β-lactams, quinolones, tetracyclines) across sources	IncFIA, IncI1, IncFII plasmids shared across sectors	WGS (illumina)	Confirms cross-source dissemination of key AMR genes and plasmids in Tanzanian One Health settings, reinforcing the mobilome perspective	Lyimo (2026)
Malawi, Uganda (Malawi subset)	Human, Animal (livestock & companion), Environment (household & wider)	*ESBL-producing Escherichia coli*	Very common ESBL genes blaCTX-M-15 (≈69% of genomes) and blaCTX-M-27 (≈15%) across 2,344 genomes, including 1814 from Malawi; dominant ST131 in Malawi	Complex network of 55 plasmids carrying blaCTX-M-15 and 30 carrying blaCTX-M-27, indicating multiple dissemination pathways and high force of selection; frequent strain and plasmid co-circulation between humans, animals, environment	WGS (illumina)	Demonstrates frequent spillover of ESBL *E. coli* between humans, animals and environment with hundreds of inferred transmission events; strongly supports need for integrated One Health genomic surveillance and WASH interventions in Malawi	Musicha et al. (177)
Malawi	Animal (food-producing poultry)—Faecal and caecal samples from broiler chickens across 10 districts (backyard farms and markets)	*Escherichia coli*	ESBL blaCTX-M genes in 48/50 cefotaxime-resistant isolates: blaCTX-M-55, −14, −65, −27, −15, −1, −3; very high resistance to tetracycline (90%) and co-trimoxazole (70%); all isolates susceptible to colistin and carbapenems	Plasmid context of blaCTX-M showed high similarity to reference plasmids from *E. coli* of various origins, suggesting shared or mobile ESBL platforms across sectors	Illumina WGS of 115 isolates plus phenotypic susceptibility testing	Reveals high AMR burden in poultry *E. coli* and ESBL plasmids related to those from other sectors, suggesting potential cross-sector ESBL flow and the need to integrate livestock into Malawi’s One Health genomic AMR surveillance	Chisembe et al. (178)
Egypt	Food chain—Dairy (milk, yogurt) and catfish from supermarkets	*Klebsiella pneumoniae*	Mostly susceptible; intrinsic blaSHV alleles and fosA variants present in all genomes	Plasmid content not fully detailed; phylogeny shows some food isolates cluster with Egyptian clinical isolates, implying shared lineages	WGS (Illumina)	Demonstrates that Egyptian food can carry *K. pneumoniae* lineages closely related to clinical strains, supporting inclusion of food sector in One Health genomic surveillance.	Anajirih et al. (179)

### Implementing one health in Africa: governance models, community delivery, and lessons learned

While genomic surveillance has advanced our understanding of AMR genes and MGEs across One Health interfaces, translating this knowledge into effective control strategies depends on how One Health is implemented in practice. Across Africa, diverse models of One Health implementation, ranging from national governance frameworks to community-centered service delivery, have emerged in response to the continent’s complex human–animal–environment interactions. This section synthesizes selected case studies from Guinea, Kenya, Ethiopia, and pastoralist communities in East Africa to examine how One Health has been operationalized, the challenges encountered, and the strategies used to address them. By comparing national platforms, institutional governance mechanisms, and community-driven approaches, we highlight key lessons that are critical for designing sustainable, context-appropriate interventions to curb the spread of AMR across ecological boundaries.

### Guinea’s regional one health platform performance

The assessment of Guinea’s regional One Health platforms provides an instructive example of the opportunities and persistent challenges associated with operationalizing One Health approaches in low-resource African settings. Guinea has established eight regional platforms designed to coordinate zoonotic disease surveillance across diverse geographic, ecological, and epidemiological zones (152). While this institutional architecture reflects strong political commitment to One Health principles, the 2025 Africa CDC assessment demonstrates that overall functionality remains limited, with a national performance score of approximately 41%, indicating that implementation has not yet reached scale or consistency across the country. A central finding of the assessment is the pronounced disconnect between policy formulation and operational capacity. Legislative and regulatory frameworks emerged as the strongest performance domain, scoring around 77%, reflecting the existence of formal policies, mandates, and coordination structures for One Health activities (152, 153). However, these policy gains have not translated into effective surveillance, preparedness, or response capacities at the subnational level. This gap illustrates a broader pattern observed in many low- and middle-income countries, where institutional frameworks are established rapidly – often following major outbreaks – without parallel investments in the resources and systems required for sustained functionality. In Guinea, this policy–practice disconnect manifests in limited inter-sectoral coordination, inconsistent surveillance activities, and weak continuity of platform operations beyond initial establishment phases.

Regional-level analysis further highlights substantial disparities in performance across Guinea’s decentralized health system. Performance scores ranged from approximately 33% in regions such as Labé and Kindia to around 46% in Faranah and Mamou, with no region achieving the 60% benchmark typically associated with functional readiness (152). These differences do not reflect isolated failures but rather systemic inequities shaped by geography, institutional capacity, and historical investment patterns. Regions that previously experienced major outbreaks, such as Ebola, or that host stronger partner engagement tended to perform slightly better, suggesting that external support and prior emergency investments continue to influence present-day capacity. Conversely, regions with limited infrastructure and weaker institutional presence struggle to maintain even basic One Health surveillance activities. Importantly, the absence of any high-performing region underscores that Guinea’s challenges are national in scope and require coordinated strategic responses rather than isolated regional interventions.

Across all regions, critical capacity gaps were evident, particularly in material resources, which scored as low as 9%. Severe shortages of basic equipment, diagnostic tools, transport, and field supplies significantly constrain the ability of platforms to conduct routine surveillance, investigate suspected outbreaks, or respond rapidly to emerging threats (154). Human resource limitations further compound these challenges. Training remains incomplete and unevenly distributed, with animal health and environmental sectors particularly affected. The lack of standardized training frameworks undermines the integration of data across sectors and limits the effectiveness of joint risk assessments and response planning. Although disease detection and registration capacities reached up to 60% in the best-performing regions, most regions remained below this threshold, highlighting persistent weaknesses in early warning systems and laboratory-linked surveillance.

These performance gaps are driven by deeper structural and systemic factors. Communication mechanisms between human, animal, and environmental health sectors remain weak, with data often collected in parallel rather than through integrated systems. Fragmented surveillance architectures limit timely information sharing and reduce the ability to detect and respond to zoonotic threats at their source. The limited adoption of digital surveillance tools, such as the Surveillance Outbreak Response Management and Analysis System (SORMAS) (155, 156), further exacerbates coordination challenges in a decentralized context where real-time data exchange is essential (Workforce instability, characterized by high staff turnover, limited career pathways, and inadequate succession planning, disrupts institutional memory and continuity, particularly at the regional level). Moreover, One Health activities lack dedicated and predictable financing, resulting in heavy reliance on short-term, donor-driven projects rather than sustained national budgetary support.

Taken together, the Guinea case demonstrates that the mere existence of One Health platforms does not guarantee functional integration or improved surveillance outcomes. Effective operationalization requires sustained investment in material resources, workforce development, and coordination mechanisms that extend beyond formal institutional establishment. The observed regional disparities further indicate that national One Health strategies must be flexible and context-sensitive, prioritizing targeted support for weaker regions while leveraging lessons from relatively better-performing areas. Strengthening digital integration, standardizing training across sectors, and embedding One Health activities into national financing frameworks are critical steps toward improving resilience and long-term sustainability.

Overall, Guinea’s experience offers broader lessons for One Health implementation across Africa. Strong policy and legislative foundations are necessary but insufficient in the absence of operational capacity, institutional coordination, and sustainable financing. Addressing these systemic constraints is essential for transforming One Health from a conceptual framework into a functional tool capable of strengthening zoonotic disease surveillance, improving outbreak preparedness, and enhancing health security in resource-limited settings (152).

### Kenya’s antimicrobial resistance national action plan (2017–2027)

Kenya’s experience in developing and implementing its National Action Plan on AMR (NAP-AMR) illustrates the evolving dynamics of One Health governance in response to a complex, multisectoral health threat (141). As AMR emerged as a pressing global and national health security concern, Kenya launched its first NAP in 2017, designed to integrate human, animal, and environmental health sectors through a One Health framework. While the plan’s adoption marked a critical policy milestone, the early years of implementation (2017–2022) exposed significant limitations that impeded progress toward strategic objectives.

The initial iteration of the NAP-AMR reflected a clear commitment to One Health principles on paper, but implementation faltered due to structural and operational shortcomings. Key activities were not adequately costed, leading to unrealistic budget estimates and misaligned resource allocation. This lack of precise financial planning undermined the feasibility of planned interventions and constrained the ability of relevant ministries and agencies to execute key program components. Compounding these challenges were unclear delineations of roles and responsibilities across sectors, which created ambiguity in accountability and dampened intersectoral collaboration (157). Crucially, the initial plan excluded important contributors to the AMR ecosystem – specifically the environment, fisheries, and plant health sectors. This omission reflected a narrower conception of One Health that did not fully encompass all reservoirs and transmission pathways for resistant organisms, ultimately weakening the plan’s comprehensiveness and effectiveness.

In response to these implementation constraints, a structured review process was instituted that leveraged both monitoring and evaluation (M&E) frameworks and extensive stakeholder consultations. This institutional learning process directly informed the revision of the strategy for 2023–2027, signaling an important shift from merely articulating One Health aspirations to embedding adaptive governance mechanisms that support implementation learning. The revised second phase of the NAP expanded sectoral coverage to include environmental, fisheries, and plant health interests, reflecting a more holistic appreciation of AMR dynamics and the need for inclusive participation across relevant domains. More realistic targets were established through sustained dialogue among sectors, and roles and responsibilities were clarified with associated accountability mechanisms, enabling clearer expectations for contribution and performance across ministries, agencies, and county governments.

An explicit outcome of this iterative governance process was the recognition of the need to strengthen coordination beyond the national level. Kenya’s constitutional framework devolves health and related functions to county governments, and the initial plan had not sufficiently accounted for this landscape of decentralized governance. The revised NAP incorporated mechanisms to foster county-level engagement and coordination, recognizing that effective AMR surveillance, stewardship, and response must be anchored not only in national strategies but also in subnational implementation structures.

The second phase of the NAP has already yielded meaningful achievements that underscore the value of these institutional adjustments. A dedicated One Health National Antimicrobial Stewardship and Infection Control (NASIC) team was established and inaugurated, serving as a focal point for cross-sectoral strategy implementation. Complementing this national body, County Antimicrobial Stewardship and Infection Control Committees (CASICs) have been developed across all 47 counties, creating structured platforms for local coordination and accountability. These county-level structures facilitate the translation of national policy into localized action, enabling surveillance, stewardship, and community engagement to be tailored to specific epidemiological and health system contexts.

Furthermore, Kenya has operationalized 22 AMR surveillance sentinel sites that routinely submit standardized data to a central data warehouse, strengthening the country’s capacity to monitor trends in resistance across human and animal health contexts (158). The development of a One Health dashboard enhances visibility of cross-sectoral trends, supporting real-time analysis and decision-making. Equally important has been the formulation of AMR stewardship guidelines adapted for both human health and animal production settings, fostering standardized practices that mitigate inappropriate antimicrobial use.

Kenya’s experience offers several lessons that are relevant for other LMICs confronting AMR through a One Health lens. First, initial iterations of national action plans are likely to reveal implementation gaps, and embedding rigorous monitoring, evaluation, and mid-course review mechanisms is essential for detecting and addressing these gaps. The iterative nature of multi-sector planning means that frameworks must be revisited and refined based on lived experience rather than remain static documents. Second, in decentralized governance systems, national strategies must explicitly articulate how coordination and accountability will function at subnational levels, recognizing that counties or regions often hold the front line of service delivery and surveillance. Third, inclusive participation from all relevant sectors, particularly those traditionally overlooked, such as environmental and plant health, is essntial from the outset to prevent costly oversights and misalignment that later demand corrective action. Finally, clear definition of roles and responsibilities, supported by accountability mechanisms, enhances sectoral ownership and reduces ambiguity that can stall implementation.

Overall, Kenya’s evolving NAP-AMR demonstrates that One Health plans must be conceived as living frameworks that evolve through practice, reflection, and adaptation.^1^ The transition from the 2017–2022 to the 2023–2027 NAP underscores the importance of responsive governance structures capable of institutional learning, and highlights that meaningful operationalization of One Health principles requires not only strategic vision but also pragmatic mechanisms for coordination, accountability, and inclusion. Kenya’s experience thus informs broader debates on how multisectoral health strategies can be effectively embedded in both national and subnational governance architectures, advancing actionable pathways toward improved antimicrobial resistance prevention and control (180).

### Ethiopia’s institutionalization of one health governance

Ethiopia’s experience with institutionalizing One Health governance illustrates the critical transition from informal coordination platforms to legally anchored, state-owned multisectoral systems. As a country facing recurrent zoonotic disease threats, high antimicrobial use in livestock, and increasing environmental pressures, Ethiopia has long recognized the relevance of One Health approaches. Prior to recent reforms, a national One Health platform existed under the Ethiopian Public Health Institute (EPHI) (159), signaling political acknowledgment of the One Health agenda. However, despite this formal presence, governance arrangements were not fully institutionalized. Coordination mechanisms across sectors were weak, lines of authority and accountability were unclear, and there was no consistent framework for allocating resources to jointly planned One Health activities. As a result, One Health collaboration remained largely *ad hoc* and project-driven, limiting its sustainability and operational impact.

The Capacitating One Health in Eastern and Southern Africa (COHESA) project^2^ played a catalytic role in addressing these structural deficiencies by grounding advocacy efforts in systematic, evidence-based assessments. Baseline evaluations conducted under COHESA identified specific governance gaps, including fragmented leadership, absence of a formalized secretariat with cross-sectoral authority, limited integration of One Health into routine institutional workflows, and weak mechanisms for joint planning and financing. Rather than introducing parallel coordination structures, the project emphasized alignment with national policy priorities and worked through existing government institutions, reinforcing domestic ownership of the reform process.

A major outcome of this approach was the enactment of Regulation No. 529/2023 by Ethiopia’s Council of Ministers, which formally established a One Health secretariat under EPHI with a clear multisectoral and multidisciplinary coordination mandate.^3^ This regulatory milestone marked a significant shift from informal collaboration to legally recognized governance, providing a durable foundation for inter-sectoral engagement across human health, animal health, environmental, and related sectors. By embedding the One Health secretariat within a national public health institution, Ethiopia strengthened the legitimacy, authority, and convening power of the One Health platform, while also clarifying institutional roles and reporting structures.

Beyond formal regulation, Ethiopia’s institutionalization process extended into strategic and operational domains. Strategic planning exercises were undertaken to improve inter-sectoral communication, harmonize planning cycles, and enhance transparency in resource management for joint One Health activities (160). These efforts aimed to address long-standing challenges in coordinating priorities across ministries with distinct mandates and budgetary processes. Capacity-building initiatives further supported the rollout of One Health functions at both national and regional levels, recognizing that effective implementation requires functional subnational structures capable of translating national strategies into local action.

An additional and distinctive element of Ethiopia’s approach was the integration of research and innovation into One Health governance. Through a “sandpit” innovation process, multidisciplinary teams were convened to co-develop novel, context-specific solutions for operationalizing One Health approaches to antimicrobial resistance. This model promoted collaboration across scientific disciplines and policy domains, reinforcing the role of evidence generation and applied research as integral components of functional One Health systems rather than peripheral academic exercises.

Several broader lessons emerge from Ethiopia’s experience that are highly relevant to other LMICs seeking to strengthen One Health governance. First, evidence-based advocacy grounded in clear identification of governance gaps can be a powerful driver of policy-level change, particularly when it proposes actionable and context-appropriate solutions. Second, government ownership is a prerequisite for sustainability; externally funded initiatives are most effective when they reinforce, rather than substitute for, national leadership and institutional priorities. Third, institutionalization must be understood as a multidimensional process encompassing legal frameworks, governance structures, resource allocation mechanisms, workforce capacity, and integration into routine operations. Addressing only one of these pillars is unlikely to yield durable impact. Finally, the establishment of new coordination mechanisms does not automatically result in functionality. Active support, continuous capacity strengthening, and sustained political commitment are essential to ensure that institutional structures translate into effective multisectoral action.

Overall, Ethiopia’s pathway toward institutionalized One Health governance demonstrates that moving from conceptual endorsement to operational reality requires deliberate, state-led reforms supported by evidence, capacity building, and alignment with national systems. The Ethiopian case underscores that One Health effectiveness depends not merely on the existence of platforms, but on their legal authority, functional integration, and ability to mobilize resources and expertise across sectors. As such, it provides a valuable model for countries seeking to embed One Health principles into enduring governance architectures capable of addressing complex challenges such as zoonotic diseases and antimicrobial resistance.^4^

### Community-centered one health delivery among pastoralist populations in East Africa

Pastoralist communities across East Africa represent one of the most compelling contexts for operationalizing One Health approaches, given the deep interdependence between human health, livestock health, ecosystems, and livelihoods (161). An estimated 268 million pastoralists inhabit approximately 40% of Africa’s landmass, predominantly in arid and semi-arid regions characterized by mobility, climatic variability, and limited access to formal services. Conventional health systems, which are typically sedentary, sector-specific, and facility-based, have historically failed to meet the needs of these populations. As a result, pastoralist communities have remained underserved, with persistent gaps in access to healthcare, veterinary services, and environmental management support.

The Health of Humans, Environment, Animals, and Livelihoods (HEAL) model^5^ emerged as an innovative response to these systemic shortcomings by reframing One Health not merely as a coordination framework at national or institutional levels, but as a community-anchored service delivery model. Implemented across pastoralist regions in Kenya, Ethiopia, and Somalia, the HEAL approach sought to align service provision with the lived realities of pastoralist communities, whose health outcomes are inseparable from livestock productivity, rangeland conditions, and mobility patterns.

Central to the success of the HEAL model was its emphasis on community-designed governance and service structures. Rather than imposing externally defined disease priorities, the model relied on Multistakeholder Innovation Platforms that enabled pastoralist communities to participate actively in identifying needs, shaping interventions, and overseeing implementation.^6^ This participatory approach fostered local ownership, ensured cultural relevance, and enhanced accountability, addressing a critical weakness of many top-down One Health initiatives that fail to resonate with community priorities. By recognizing pastoralists as co-producers of health rather than passive beneficiaries, the HEAL model strengthened trust and sustained engagement over time.

Operational flexibility was another defining feature of the HEAL approach. Service delivery combined both mobile and static One Health Units, allowing interventions to adapt to seasonal migration, grazing patterns, and climatic shocks. Mobile units were particularly important for reaching remote and transient populations, while fixed units provided continuity in areas of seasonal settlement. This hybrid delivery model directly addressed one of the structural barriers to healthcare access in pastoralist settings: the mismatch between mobile populations and immobile service infrastructure.

At the core of HEAL’s implementation was the deployment of genuinely multisectoral teams composed of government-employed professionals from human health, veterinary services, environmental management, and natural resource sectors. By working side by side at the community level, these teams operationalized One Health integration where it matters most – at the point of service delivery. Co-location and joint problem-solving not only improved efficiency but also fostered professional relationships and mutual understanding across sectors that are often siloed at higher administrative levels. This bottom-up integration contrasts with many One Health initiatives that emphasize coordination at national policy forums while leaving frontline service delivery fragmented.

The HEAL model also demonstrated the importance of embedding interventions within a broader socio-ecological understanding of pastoralist systems. Health challenges in these settings cannot be disentangled from land use, water access, herd management, and environmental degradation. By integrating rangeland management and land-use planning into health service delivery, the model addressed upstream determinants of both human and animal health. This approach contributed to improved stewardship of shared natural resources, reinforcing the reciprocal relationship between environmental sustainability and community well-being.

Observed outcomes across the three countries included improved access to integrated health services in remote pastoralist areas, enhanced collaboration between human and animal health workers, and evidence of strengthened community trust in public institutions. Importantly, these gains were not short-lived. A long-term funding commitment spanning more than a decade, combined with strong government ownership, allowed the HEAL model to evolve, adapt, and embed itself within national and subnational systems. This temporal dimension is particularly significant, as many One Health initiatives fail to achieve sustainability due to short project cycles and limited institutional integration (162).

Several broader lessons emerge from the HEAL experience that are highly relevant for One Health implementation in LMICs. First, One Health interventions are most effective when they are driven by community priorities rather than externally defined disease agendas. Second, integration is most tangible and impactful at service delivery points, where co-located, multisectoral teams can build trust, shared practice, and collaborative problem-solving. Third, a deep understanding of socio-ecological systems is essential for designing interventions that are relevant, acceptable, and sustainable. Finally, long-term investment and government ownership are critical enablers of success, allowing One Health models to move beyond pilot phases into durable systems of care.

Overall, the HEAL model demonstrates that One Health can be effectively operationalized at the community level when governance, service delivery, and cultural context are aligned. In pastoralist settings, where health, livestock, and the environment are inseparable, community-centered One Health delivery offers a powerful pathway for reducing inequities, strengthening resilience, and translating One Health principles into meaningful and sustained impact (DOI: 10.1079/onehealthcases.2024.0004).

## Conclusion and prospects

This review highlights that plasmids from broad-host-range and clinically important families, together with MGEs, are central drivers of AMR transmission across human, animal, and environmental compartments. At the same time, AMR in Africa cannot be addressed through technical interventions alone. Its emergence and persistence are shaped by several structural and systemic drivers as discussed. Also, despite growing advances in One Health–oriented genomic studies in Africa, important knowledge gaps remain. These include limited understanding of plasmid ecology and the dynamics of MGEs in natural and anthropogenically impacted environments, insufficient characterization of environmental resistomes across diverse ecological niches, and a lack of longitudinal, high-resolution genomic surveillance capable of tracking AMR evolution and transmission over time. Addressing these gaps will be essential for improving predictive capacity and strengthening evidence-based One Health interventions across the continent. The One Health approach therefore remains essential, not only as a conceptual framework, but as an operational strategy for integrating these sectors in surveillance, prevention, and response. Experiences from African countries including Guinea, Kenya, and Ethiopia, as well as community-based One Health initiatives in pastoralist settings, demonstrate that multisectoral coordination platforms, integrated surveillance systems, and cross-sector engagement can be established even in resource-constrained contexts. However, these experiences also show that implementation remains uneven and often limited by insufficient financing, weak interoperability between data systems, constrained laboratory and bioinformatics capacity, and poor translation of surveillance findings into policy and practice. Taken together, the evidence in this review suggests that the next phase of One Health AMR research and control in Africa should move beyond descriptive genomic studies toward coordinated, policy-relevant genomic surveillance systems. Several priorities emerge from this review.

First, minimum data standards are needed for One Health genomic surveillance across the continent. Genomic data should be accompanied by standardized metadata covering isolate source, host or environmental compartment, geographic location, date of sampling, antimicrobial susceptibility phenotype, relevant antimicrobial exposure history where available, and key plasmid and MGE annotations. Harmonized metadata standards would improve comparability across studies, support continental-scale analyses, and strengthen the value of public genomic repositories for tracking AMR dissemination. Second, sampling frameworks should be harmonized across sectors and designed longitudinally rather than opportunistically. Much of the current evidence is based on cross-sectional and uneven sampling, which limits inference on transmission dynamics. Future surveillance should integrate synchronized sampling from humans, animals, food systems, wastewater, surface waters, farm environments, and healthcare settings, with repeated sampling over time to capture persistence, seasonal variation, and possible plasmid or clone turnover. Such designs are essential for identifying transmission interfaces, distinguishing transient contamination from sustained circulation, and detecting emerging high-risk resistance platforms.

Third, environmental surveillance must become a routine component of One Health AMR genomics in Africa. Environmental compartments remain under-sampled despite their central role as reservoirs, mixing grounds, and dissemination pathways for resistant pathogens, plasmids, and resistance genes. Wastewater treatment plants, hospital effluents, abattoir waste streams, surface waters, irrigation systems, soils, and aquaculture environments should be systematically incorporated into surveillance frameworks. This is particularly important for understanding the role of non-clinical selection pressures, in maintaining and amplifying resistance and mobile resistance elements.

Fourth, sequencing and laboratory capacity must be strengthened through regional and national investment. Although genomic studies are increasing across Africa, access to sequencing platforms, quality-assured laboratory workflows, and sustainable reagent supply chains remains uneven. Strengthening infrastructure should include support for DNA extraction and library preparation workflows, access to short- and long-read sequencing where feasible, quality control pipelines, and training in genomic sequencing. Building distributed sequencing capacity through regional hubs linked to national laboratories may offer a practical and scalable model for countries where stand-alone national capacity is still developing. Also, bioinformatics infrastructure and workforce development require urgent expansion. Sequencing alone will not translate into actionable surveillance without robust analytical capacity. Investment is needed in high-performance computing access, secure data storage, curated reference databases, reproducible analysis pipelines, and training in comparative genomics, phylogenomics, AMR gene and plasmid analysis. Importantly, bioinformatics strengthening should not be treated as an optional downstream component, but as a core pillar of genomic surveillance systems.

Furthermore, there is need to formalize cross-sectoral data sharing and governance mechanisms. Fragmented data systems currently limit the ability to link genomic findings across clinical, veterinary, food, and environmental sectors. National and regional One Health AMR platforms should support interoperable databases, common reporting structures, and clear governance frameworks for data ownership, access, and sharing. Linking genomic data with antimicrobial use, resistance phenotypes, epidemiological metadata, and environmental measurements would significantly improve interpretation of transmission patterns and co-selection pressures. Also, genomic evidence must be translated more directly into policy and AMR control interventions. Surveillance systems should be designed not only to generate genomic data, but also to inform antimicrobial stewardship, infection prevention and control, wastewater and farm waste management, outbreak investigation, and risk assessment for high-risk clones, plasmids, and resistance genes. This requires stronger interfaces between researchers, public health agencies, veterinary authorities, environmental regulators, and policymakers, as well as clear mechanisms for converting genomic findings into actionable guidance and intervention priorities.

Finally, the central message from this review is that One Health genomic surveillance in Africa must be both standardized and context-responsive. Core surveillance principles, minimum metadata requirements, and analytical standards should be harmonized across countries, but implementation models must remain adaptable to local ecological, socio-economic, infrastructural, and cultural realities. A one-size-fits-all model is unlikely to succeed across the continent’s highly diverse health systems and production environments. The most effective path forward is therefore a pragmatic One Health strategy that combines shared continental standards with locally tailored surveillance design, sustained financing, and robust political commitment. Overall, controlling AMR in Africa will require more than documenting resistance genes and resistant organisms in isolated sectors. It will require integrated genomic surveillance systems capable of translating those findings into coordinated public health, veterinary, agricultural, and environmental action.

## Footnotes

1
https://www.who.int/publications/i/item/9789240062689

2
https://www.ilri.org/research/projects/capacitating-one-health-eastern-and-southern-africa-cohesa

3
https://ephi.gov.et/wp-content/uploads/2023/11/Ethiopia-Health-Security-Bulletin-Sep.-2023.pdf

4
https://ephi.gov.et/wp-content/uploads/2025/09/One-Health-Strategic-Plan-FINAL.pdf

5
https://www.oh4heal.org/

6
https://www.oh4heal.org/heal/
